# Vaccination in preterm and low birth weight infants in India

**DOI:** 10.1080/21645515.2020.1866950

**Published:** 2021-02-18

**Authors:** Santosh Soans, Attila Mihalyi, Valerie Berlaimont, Shafi Kolhapure, Resham Dash, Ashish Agrawal

**Affiliations:** a Paediatrics, AJ Institute of Medical Sciences, Mangalore, India; b Medical Affairs and Clinical R&D, GSK Vaccines Europe, Wavre, Belgium; c Global Medical Affairs, GSK, Wavre, Belgium; d Medical Affairs Department, GSK, Mumbai, India; e Medical Affairs Department, GSK, Bengaluru, India; f Medical Affairs Department, GSK, Hyderabad, India

**Keywords:** Neonatal, preterm, low birth weight, immunization, India, prematurity, infectious disease, vaccination

## Abstract

In India, the high neonatal and infant mortality rate is due in part to an increasing number of preterm and low birth weight (LBW) infants. Given the immaturity of immune system, these infants are at an increased risk of hospitalization and mortality from vaccine-preventable diseases (VPDs). In this narrative review, we screened the scientific literature for data on the risk of VPDs, vaccination delay and factors related to it in Indian preterm and LBW infants. Although routine childhood vaccinations are recommended regardless of gestational age or birth weight, vaccination is often delayed. It exposes these infants to a higher risk of infections, their associated complications, and death. After-birth complications, lack of awareness of recommendations, vaccine efficacy and effectiveness and concerns related to safety are some of the common barriers to vaccination. Awareness campaigns might help substantiate the need for (and value of) vaccination in preterm and LBW infants.

## PLAIN LANGUAGE SUMMARY

What is the context?

- In India, the high neonatal mortality rate is due in part to an increasing number of pretern and low birth weight intants.
- Affected infants have a poorly developed inmune system and are more susceptible to contracting vaccine-preventable diseases.
- The Indian Academy of Pediatrics recommends vaccination according to the same schedule used for full term infants, following chronological (not gestational) age.
- Delays in vaccinations increase the risk of preventable infections.

What is new?

- Our review of the scientific literature shows that, in India:
  - infections have more serious conseuences in preterm and low birth weight infants
  - delays to vaccinate affected infants are common, mostly due to safety and effectiveness concerns from parents and healthcare pracitionrs.

What is the impact?

- Improving mternal nutritional status and immunization, and perinatal care could help reduce the number of preterm and low birth weight infants.
- Combining maternal immunization with vaccination of affected infants can confer safe and effective protection.
- Awareness campaigns for parents and healthcare practitioners could address the issue of vaccination delay in pretern and low birth weight infants in India.

## Introduction

Preterm birth and low birth weight (LBW) in newborns is a source of significant global public health concern.^1^ Although LBW often characterizes preterm babies, the two terms cannot be used interchangeably.^2^ World Health Organization (WHO) defines preterm newborn as birth before 37 weeks of gestation and further categorizes it into extremely preterm (<28 weeks), very preterm (28–32 weeks) and moderate to late preterm (32–37 weeks).^3^ LBW is defined as weight at birth of <2,500 g and is categorized into very LBW (<1,500 g) and extremely LBW (<1,000 g).^4^

Preterm birth can be either spontaneous or induced (e.g. elective cesarean or other non-medical reasons).^5,6^ Correspondingly, LBW could be associated with preterm birth, or could be due to restricted fetal growth, or a combination of both.^7^ Risk factors for prematurity and LBW include undernutrition, genetics, infections, underlying comorbidities (e.g., diabetes), maternal history of multiple pregnancies, chronic maternal stress induced by infections and inflammation, socioeconomic factors, and lifestyle choices of the mother (e.g., smoking).^6,8^

Preterm and LBW infants are at a higher risk of infections and death compared to full-term and normal birth weight infants.^9^ The major risk factors are perinatal infections, prolonged hospitalization after birth, iatrogenic complications of lifesaving therapies, low levels of circulating maternal antibodies, and an immature immune system.^9^ Specifically, the immaturity of the immune system is known to increase with decreasing gestational age and birth weight.^10–12^ Perinatal infections could be fatal and are associated with long-term sequelae that can lead to impaired neuro-developmental functioning, inhibited growth, chronic diseases and long-term physical health consequences.^6,10–12^

Increasing numbers of preterm and LBW newborns every year could add to the disease burden on healthcare systems and individual families, depending on the setting.^1,6,8,13^ According to the WHO, more than 10% of infants (i.e., ~15 million infants per year) were born preterm and 15%–20% of infants (i.e., >20 million infants per year) were born with LBW in 2014–2015.^1,2,14^ Preterm birth directly contributes to neonatal mortality, accounting for nearly 1 million deaths every year,^1^ while LBW is a major predictor of mortality and morbidity in preterm children.^1^ Highest levels of neonatal mortality and morbidity are reported in low- and middle-income countries, with Africa and Asia being responsible for the majority of this public health burden.^15,16^ In 2018, approximately 50% of all deaths under 5 years of age were reported from just five countries: Democratic Republic of the Congo, Ethiopia, India, Nigeria, and Pakistan. Among these countries, about 33% of deaths were reported in Nigeria and India alone.^17^

## The Indian context

In 2017, India recorded approximately one million deaths (20% of the global) among children under 5 years of age.^18^ Of these, 0.57 million were neonatal deaths in which the reported causes were preterm birth (27.7%), encephalopathy due to birth asphyxia and trauma (14.5%), lower respiratory infections (11.0%), congenital birth defects (8.6%), sepsis and other infections (6.1%), hemolytic disease and jaundice (3.2%), diarrheal diseases (2.7%), tetanus (0.7%), other disorders (22.0%), and other causes (3.5%).^18^ This situation is alarming as India accounts for 23.4% of the global preterm births.^14^ Estimates of LBW infants are notable: during 2013–2014, amongst approximately 19 million newborns,^19^ 68.7% were weighed at birth and among these, 18.6% were LBW (i.e., approximately 2.43 million births).^20^

The majority of deaths in children under 5 years of age and morbidity associated with infectious diseases can be averted by timely interventions including adequate nutrition, clean water, appropriate maternal care during pregnancy and immunization of the mother and infant.^17^ The WHO and the Advisory Committee on Vaccines and Immunization Practices of the Indian Academy of Pediatrics (IAP) recommend that all infants receive immunization, regardless of any restrictions based on gestational age or birth weight, with the qualified exception of the hepatitis B vaccine as the birth dose is not counted toward the full schedule due to a reduced immune response.^21–23^ Table 1 provides an overview of the recommended vaccines in children ≤12 months of age.

**Table 1.** Vaccination recommendations and overview of availability of immunogenicity and safety of recommended vaccinations for preterm and LBW infants ≤12 months of age

									Immunogenicity/Effectiveness		Safety	
Vaccine^₶^	Recommended by WHO^24^	Europe (ECDC)^Ɣ25^	USA (CDC)^26,27^	Canada^28^	Australia (ATAGI)^Ƙ29^	India (IAP)^21^	Included in the Indian NIP^22^	Age (chronological age) recommendation in India^21,22^	Preterm (<37 weeks) ^±^	LBW (<2,500 grams) ^±^	Preterm (<37 weeks) ^±^	LBW (<2,500 grams) ^±^
BCG	Yes	Yes^$^	n.a	n.a	n.a	Yes	Yes	At birth or as early as possible till one year of age	Yes^30^	Yes^30^	Yes^30^	Yes^30^
Hepatitis B	Yes	Yes	Yes	Yes	Yes	Yes	Yes	First dose at birth or as early as possible within 24 hours	Yes^31,32^	Yes^23,31^	Yes^31,32^	Yes^23^
OPV – 0	Yes	n.a	n.a	n.a	n.a	Yes	Yes	At birth or as early as possible within the first 15 days	Yes^33^	n.a.	Yes^33^	n.a.
OPV – 1, 2, 3	Yes	n.a	n.a	n.a	n.a	Yes, At 6 weeks and 14 weeks, in case IPV not available or feasible	Yes	At 6 weeks, 10 weeks and 14 weeks (OPV can be given till 5 years of age)	Yes^34^	n.a.	Yes^34^	n.a.
Fractional IPV	Yes	Yes	Yes	n.a	n.a	Yes	Yes	Two fractional doses at 6 and 14 weeks of age	Yes^35,36^	n.a.	Yes^36^	n.a.
Pentavalent combination (DTaP-Hib-Hep B)	n.a	Yes	n.a	n.a	n.a	Yes	Yes	At 6 weeks, 10 weeks and 14 weeks (can be given till one year of age)	n.a.	n.a.	n.a.	n.a.
Hexavalent combination (DTaP-Hib-IPV-Hep B)	n.a	Yes	n.a	Yes	Yes		No	n.a.	Yes^37,38^	Yes^37,38^	Yes^37,38^	Yes^37,38^
PCV	Yes	Yes	Yes	Yes	Yes	Yes	Yes (select states)	At 6 weeks and 14 weeks. Booster dose at 9–15 months of age.	Yes (PCV7, PCV10, PCV13)^39–41^	Yes (PCV7, PCV10)^39,40^	Yes^41^	Yes^39^
Rotavirus	Yes	Yes	Yes	Yes	Yes	Yes	Yes (select states)	At 6 weeks, 10 weeks & 14 weeks (can be given till one year of age)	Yes^42–45^	n.a.	Yes^42–45^	n.a.
Influenza	No	No	n.a	Yes	Yes	Yes	No	6 months–5 years	Yes^46^	Yes^46^	Yes^46^	Yes^46^
Measles, mumps, rubella, varicella^£^	n.a	Yes	Yes	Yes	Yes	Yes	Yes	9 months-12 months	n.a.	n.a.	n.a.	n.a.

^₷^Vaccinations should be administered to preterm and LBW infants according to the recommended schedule at the discretion of the physician

^Ɣ^The data presented in the Table is a comparison of recommendations for the UK and Germany

^Ƙ^For preterm infants only

^±^Limited evidence in infants with a gestational age of<31 weeks and very LBW infants

^$^For infants in areas of the country with TB incidence ≥ 40/100,000. Infants with a parent or grandparent born in a high incidence country

^£^Individual vaccine recommendations are reported for measles, mumps, rubella and varicella vaccines and not combination vaccines

ATAGI: Australian Technical Advisory Group on Immunization; BCG: Bacillus Calmette-Guerin vaccine; CDC: Centers for Disease Control and prevention; ECDC: Europe Centre for Disease prevention and Control; IAP: Indian Academy of Pediatrics; NIP: National Immunization Program; LBW: low birth weight; TB: tuberculosis; OPV: oral polio vaccine; IPV: inactivated polio vaccine; DTaP: diphtheria, tetanus, pertussis vaccine; Hib: *Hemophilus influenza* type b; Hep B: hepatitis B; PCV-7, −10, −13: pneumococcal conjugate vaccine, −7 valent, −10 valent and −13 valent; MR: measles and rubella; MMR: measles, mumps, rubella; MMRV: measles, mumps, rubella with varicella; n.a.: not available; WHO: World Health Organization

## Rationale of the review

Despite the existence of vaccination recommendations, several studies in high-income countries have reported either a significant delay or a complete lack of immunization in preterm infants.^47–50^ The situation is unlikely to be different in India, as a high level of vaccine-preventable disease (VPD) burden in infants or children persists.^51^ Within this context, there is a need to better understand the factors and barriers related to the absence or delay in vaccination among preterm and LBW infants. This information could help bridge existing knowledge gaps in the scientific community, specifically among healthcare providers (HCPs) who are perceived as the most trusted advisors and influencers of vaccination decisions.^52^

**Figure 1.**
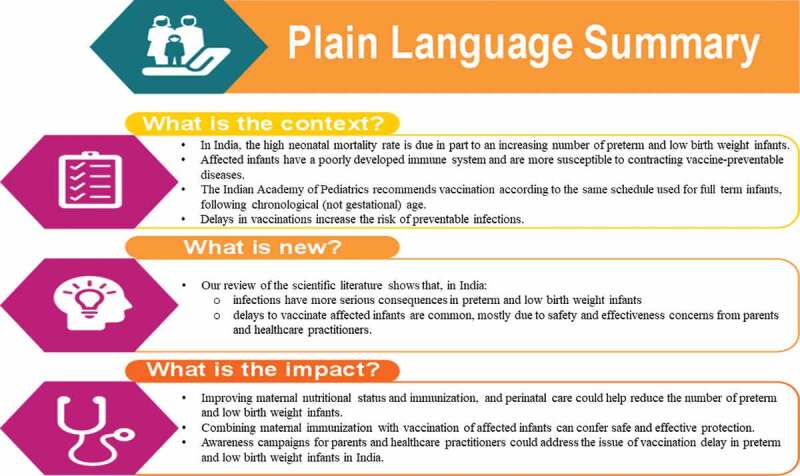
Plain language summary.

A recent publication summarizing practical issues surrounding vaccination in preterm infants lends support to the implementation of existing vaccination recommendations for preterm and LBW infants in India.^53^ However, information on the extent of vaccination delay in preterm and LBW infants has not been previously summarized. In this review, we outline the rationale for immunization and highlight the risks of VPDs in preterm and LBW infants. We also provide an overview of recommended vaccinations, with a focus on whether efficacy/effectiveness and safety data are available in these populations. Lastly, we present the caveats linked to different vaccination strategies that could be utilized to mitigate the burden of VPDs in preterm and LBW infants in India. Figure 1 elaborates on the findings in a form that could be shared with patients by HCPs.

## Characteristics of the immune system of preterm and LBW infants

Neonates predominantly rely on their first line of defense (physical barrier) and then innate immune response rather than adaptive immune response. At birth, both immune defense mechanisms are immature.^54^ This immune system immaturity is amplified in infants born preterm and in those with LBW, due to several deficiencies (Table 2).

**Table 2.** Characteristics of the immune response in preterm and LBW infants^10^^,^^54^^–^^56^^.^

Type of immune response	Role at nominal level	Characteristics in preterm and LBW infants
**Innate immunity**		
Soluble proteins and peptides^10,56^	Able to opsonize pathogens thus aiding phagocytosis and kill pathogens with their antimicrobial properties	Limited production leads to preterm infants experiencing limited exposure to breast milk that contains antimicrobial peptides
IgG	Protective antibody against viruses, bacteria and anti-toxins	Limited production Low levels of maternal IgG (increases with fetal age)
APPs^10,56^	Destroy pathogens via diverse mechanisms	Reduced production in preterm infants and reduced exposure through breast milk
BPI^55^	Neutralizes the lipopolysaccharide endotoxin and is cytotoxic to gram negative bacteria	Found at lower concentrations in preterm infants
NK cells^55^	Produce cytotoxicity and lyse infected cells or antibody-sensitized cells	Lower activity Reduced number of cells compared to full-term infants
Classical, alternative and lectin complement pathways^54–56^	MBL is a well characterized activator of the lectin pathway in antibody deficient neonates L-ficolin is a major pattern recognition molecule involved in activation of the lectin pathway	Reduced pathogen-killing abilities Deficient in production of C1, C4 (classical pathway) and factor B (alternative pathway) Deficient in pattern-recognition receptor MBL MBL could be associated with particular deleterious effects in preterm infants Low production of GM-CSF and G-CSF hormones L-ficolin production and function reduced
Phagocytes^54^	Include neutrophils, monocytes and macrophages	Limited chemotaxis of phagocytes to infectious sites
Neutrophils^10,55^	Phagocytose and destroy microorganisms intracellularly by utilizing a variety of toxic substances	Have reduced pool including precursors due to reduced glycoprotein hormones G-CSF and GM-CSF difficulty in migrating to sites of infection due to reduction in expression of adhesion molecules such as L – and *P*-selectin Decreased phagocytic activity of preterm neutrophils Preterm neonates have decreased respiratory burst Reduced expression and inducibility of integrins or arrest of cell-rolling and adhesion
Monocytes^10,55,56^	Phagocytose and destroy microorganisms intracellularly by utilizing a variety of toxic substances Differentiate into macrophages or dendritic cells in tissue	Smaller pool of monocytes due to reduced glycoprotein hormones G-CSF and GM-CSF Reduction in cytokine production Low levels of chemotaxis and phagocytosis during infection Reduced ability of costimulatory molecules upregulation Limited capacity to activate B – and T-cells Adaptive immune response activation is limited
**Adaptive immunity**		
Overall	-	Maturation of this type of immunity occurs mostly after term birth All newborns have deficiencies in T-cell activation, cytokine production, cytolytic activity, B cell immunoglobulin production, and cooperation between T and B-cells
Circulating lymphocytes (B and T-cells)^10,54^	Produce or express cytokines and IgM antibodies	Lower absolute numbers Lower numbers of naïve T – and B-cells Lower IgG concentrations
T lymphocytes helper (CD4+)^55^	Respond to new antigens by producing or expressing cytokines in their cell membrane Activated by MHC class 2	Decreased stimulation of B-lymphocytes to produce antibodies
Cytotoxic lymphocytes (CD8+)^55^	Eradicate lysed cells through clonal expansion of antigen-specific cytolytic cells Activated by MHC class 1	Decreased cytotoxic activity Lower absolute numbers of CD3 and CD8 cells

APP: antimicrobial proteins and peptides; BPI: bactericidal permeability increasing protein; CD: cluster of differentiation; IgG: immunoglobulin G; IgM: immunoglobulin M; LBW: low birth weight; MBL: mannose-binding lectin; G-CSF: granulocyte colony-stimulating factor; GM-CSF: granulocyte-macrophage colony-stimulating factor; MHC: major histocompatibility complex; NK: natural killer

Defense against pathogens consists of physical barriers, such as keratinized skin and mucous membranes lining the respiratory and gastrointestinal tracts, and chemical barriers containing various enzymes and other substances that elicit a direct antimicrobial action or inhibit microbial adherence to body surfaces.^55^ Compared to full-term infants, this barrier is undeveloped in preterm and LBW infants, making it susceptible to ruptures and therefore serving as an inefficient defense barrier.^55^ Furthermore, antimicrobial peptides-producing flora are reduced in number within the mucosal barrier of the respiratory and gastrointestinal tracts, thus facilitating the penetration of pathogens and increasing the risk of infection.^57^

When pathogens cross the first line of defense, the innate immune response is triggered through several pathways. This innate immune response is partial in preterm and LBW infants due to availability of smaller number of neutrophils compared to full-term and normal birth weight infants. Neutrophils generate oxygen radicals that facilitate intracellular killing of pathogens and can also perform phagocytosis.^55,56,58^ Similarly, a smaller pool of monocytes is available in preterm and LBW infants. Monocytes are capable of phagocytosis, secretion of cytokines or chemokines and antigen presentation, and regulate the activation of B-cells and T-cells, which are integral parts of the adaptive immune response.^55,56^ Consequently, preterm and LBW infants are at a high risk of infection (Table 2).

Intrauterine inflammation, which may cause premature immune activation and cytokine production, directly contributes to preterm birth,^56^ and may lead to immune tolerance and reduced immune function in preterm and LBW newborns. Furthermore, medical interventions at the time of delivery can impact immune development and function. For example, antenatal corticosteroid treatment to prevent newborn respiratory disease is associated with reductions in lymphocyte proliferation, cytokine production and an increased risk of infection.^56^

Soluble proteins such as immunoglobulins (Ig) and peptides facilitate phagocytosis and elicit antimicrobial properties. The production of soluble proteins by the fetus is limited and thus adaptive immunity is mostly provided through maternal antibodies. Maternal IgG antibodies are transferred to the fetus starting at approximately 17 weeks of gestation, with cord-blood IgG levels similar to maternal titers after 32 weeks of gestation and up to 2-fold higher at term birth.^59,60^ Due to this, preterm infants have low levels of circulating maternal IgG as a function of gestational age at birth. This leads to a higher susceptibility of infants to contract infections, including those that can be prevented by vaccinations.^59,61^

## VPDs in preterm and LBW infants

**Figure 2.**
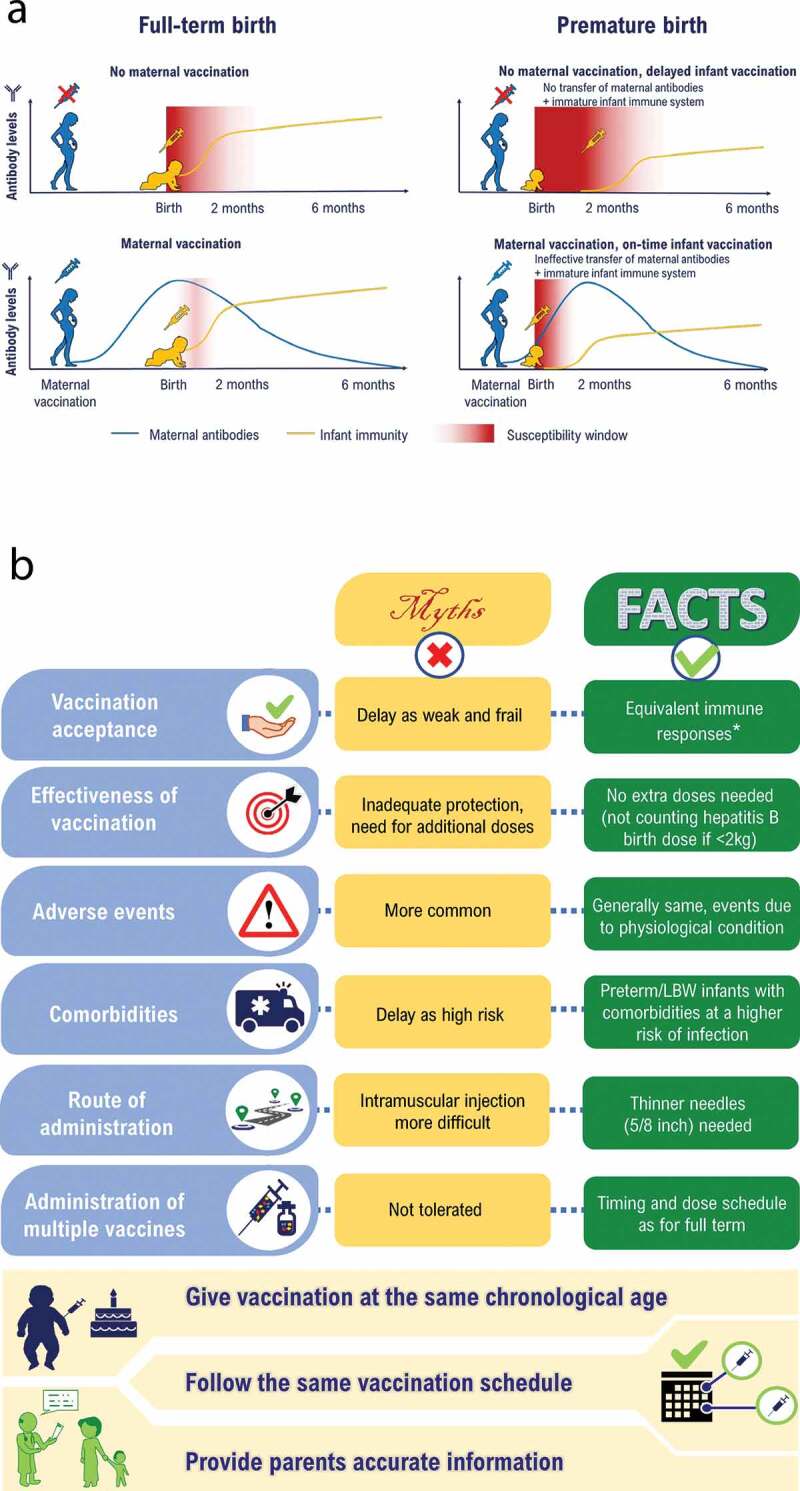
Vaccination in preterm and LBW infants in India (A) Window of susceptibility to disease (B) Barriers to vaccination due to knowledge gaps among HCPs and parents ^± ‡^.

Newborns usually contract infections either in the perinatal or the postpartum period. Exposure to infections is especially critical in preterm and LBW infants because of their immature immune system and inadequate levels of maternal antibodies.^54–56,59^ This aspect is depicted in Figure 2A for reference. Data on the risk of VPDs among preterm and LBW infants in India are lacking, therefore we report information from other relevant countries (Table 3). In comparison to full-term and normal birth weight infants, preterm and LBW infants are at an increased risk of hospitalization and mortality from VPDs such as diphtheria,^62^ influenza,^68^ invasive pneumococcal disease,^39^ bacterial meningitis,^66^ pertussis,^64,65,69^ bacterial and viral pneumonia,^66^ rotavirus gastroenteritis^67^ and tetanus.^63^ Importantly, the literature suggests that an increased risk of infection positively correlates with the degree of prematurity and LBW.^66,70^ Specifically, infection of the very and extremely LBW infants with opportunistic and aggressive multidrug-resistant pathogens often results in death.^70^

**Table 3.** Risk of vaccine-preventable disease in preterm and LBW infants

Disease	Outcome	Risk of acquiring disease
Diphtheria	Increased risk^Ƙ^ of disease^62^	n.r.^¥^
Tetanus	Death due to neonatal tetanus in LBW	OR: 2.09 (95%CI: 1.29–3.37)^63^
Pertussis	Hospitalization in preterm vs. full-term infants	IRR: 1.99 (95%CI: 1.47–2.71)^64^
	Severe disease with a history of prematurity	OR: 5.00 (95%CI: 1.27–19.71)^65^
Polio	Increased risk of disease	n.r. ^¥^
Hepatitis B	Increased risk of disease	n.r. ^¥^
Invasive pneumococcal disease	Risk of infection in LBW (<2,500 grams) infants	RR: 2.6 (*P* = .03)^39^
	Risk of infection in preterm (<38 weeks) infants	RR: 1.6 (*P* = .06)^39^
Bacterial meningitis	Hospitalization in infants weighing <1,000 grams Hospitalization in infants weighing 1,000–1,499 grams Hospitalization in infants weighing 1,500–1,999 grams Hospitalization in infants weighing 2,000–2,499 grams	RR: 1.38 (95%CI: 0.57–3.35)^66^ RR: 1.46 (95%CI: 0.88–2.44)^66^ RR: 1.55 (95%CI: 1.13–2.12)^66^ RR: 1.31 (95%CI: 1.09–1.58)^66^
Bacterial pneumonia	Hospitalization in infants weighing <1,000 grams Hospitalization in infants weighing 1,000–1,499 grams Hospitalization in infants weighing 1,500–1,999 grams Hospitalization in infants weighing 2,000–2,499 grams	RR: 2.86 (95%CI: 1.83–4.47)^66^ RR: 1.67 (95%CI: 1.20–2.33)^66^ RR: 1.53 (95%CI: 1.22–1.91)^66^ RR: 1.51 (95%CI: 1.32–1.71)^66^
Rotavirus gastroenteritis	Hospitalization in very LBWs (<1,500 grams)	OR: 2.6 (95%CI: 1.6–4.1)^67^
	Hospitalization in LBW (1,500–2,499 grams)	OR: 1.6 (95%CI: 1.3–2.1)^67^
Influenza	Severe disease in children with history of prematurity	OR: 2.53 (95%CI: 1.34–4.77)^68^

^Ƙ^Age data reflect a higher proportion of cases in the adolescent and adult populations. These populations could be the source of infection in preterm and LBW infants

^¥^No data was found for these diseases. It can be assumed that there is a high risk of these diseases occurring given the immaturity of the immune system of the preterm and LBW infant

CI: confidence interval; IRR: incidence rate ratio; LBW: low birth weight; OR: odds ratio; P: *p*-value; RR: relative risk

*Immune response refers to sero-conversion/sero-protection levels^±^Vaccination recommendations in the National Immunization Programme (Government of India)^22^ and Indian Academy of Pediatrics (optional schedule)^21^ is provided in Table 1 ^‡^Panel B was created from Table 1 of Sahoo et al. 2020,^53^ and the personal opinions of the authors of this manuscript; HCP: healthcare professional; LBW: low birth weight

## Vaccination programs and timing in preterm and LBW infants

**Figure 3.**
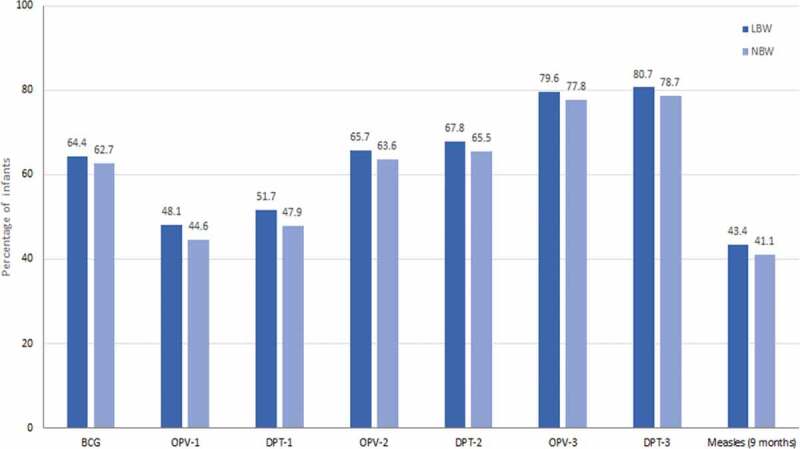
Immunization delay among LBW infants^±‡^.

^±^Statistically significant difference (*P* < .05) compared to normal birth weight infants was documented for all vaccines. Vaccination was administered according to the National Immunization Programme (Government of India). BCG and OPV-0 at birth, OPV-1/DPT-1 at 6 weeks of age, OPV-2/DPT-2 at 10 weeks of age, OPV-3/DPT-3 at 14 weeks of age and measles at 9 months of age.^21,22^ Delayed vaccination for each vaccine was defined as administration of the vaccine dose after 28 days (i.e. 4 weeks) of the minimum recommended age, as per the National Immunization Programme (Government of India).^22^

^‡^Created from Table 2 of Upadhyay et al. 2017.^73^ The reported data were obtained from the rural Haryana region BCG: Bacillus Calmette-Guerin vaccine; DPT: diphtheria, pertussis, tetanus vaccine; OPV: oral polio vaccine; LBW: low birth weight; NBW: normal birth weight

Published literature suggests that vaccination in preterm and LBW infants is delayed despite the existence of recommendations.^47–50^ Due to this, the risk of complications and mortality from preventable infections is multiplied as the susceptibility window to infections is increased from the time of birth.^69^ Vaccination delay or refusal of vaccines for preterm and LBW infants appears to be a prevalent issue in India as documented from several studies.^71–73^ In these studies, delays in timely vaccination for each vaccine was defined as administration of the vaccine dose after 28 days of the minimum recommended age, meaning that vaccination was categorized as delayed if given on day 29 or later for Bacillus Calmette-Guerin (BCG), 71 days or later (after 10 completed weeks) for diphtheria-pertussis-tetanus (DPT)-first dose (DPT-1) and for DPT–third dose (DPT-3), when the infant was vaccinated at >18 weeks of age.^71–73^ For measles, delayed vaccination was defined as having received the vaccine after 4 weeks of recommended/due-time, i.e. after 9 completed months of age (measles is recommended at 9 months of age).^74^ In the first prospective study, almost half of the infants <33.5 weeks of gestational age (very preterm) and weighing <1,500 g (very LBW) were without immunization, while 62.5% of the remaining infants had a documented delay in immunization.^72^ In the second study, data from the National Family and Health Survey-4 revealed that LBW infants with a birth weight <2,000 g had higher odds of a delay in receiving the BCG vaccine (adjusted odds ratio [aOR] 2.33, 95% confidence interval [CI] 1.89, 2.89) and the DPT-1 (aOR 1.53, 95% CI 1.26, 1.86) and the first dose of the measles vaccine (aOR 1.36, 95% CI 1.11, 1.67).^71^ In a third study, in which 10,644 LBW infants (<2,500 g) were enrolled and followed until 12 months of age, a significantly lower immunization uptake was documented both in terms of the proportion of infants immunized and of the timing of vaccine administration (Figure 3). About 3 out of 10 LBW infants were fully immunized by the age of 1 year (i.e., had received the BCG vaccine, three doses of the DPT vaccine, the oral polio vaccine, and the measles vaccine).^73^ There was a delay in the time of administration of the vaccines compared to the recommended timing. The median delay (interquartile range) for the BCG vaccine was 41 (19–75), and for the three doses of the DPT vaccine (DPT–1, DPT–2 and DPT–3) was 30 (12–63), 46 (23–89) and 62 (34–112) days, respectively.^73^ For the measles vaccine, the median delay from the recommended timing was 24 (9–46) days.^73^

## Barriers to vaccination in preterm and LBW infants

Overall in India, several barriers to infant vaccination according to the recommended schedule have been documented. Vaccine hesitancy was a common barrier across different age groups.^52,75,76^ Several factors were identified as causes of vaccine hesitancy in India: these relate to immunization effectiveness, safety/adverse events, provider belief, attitudes of parents, religious/socioeconomic factors, and policy guidelines regarding vaccination.^52,75^ These factors become even more complex in preterm and LBW infants.^53,71–73^ Factors of delayed vaccination in preterm and LBW infants were identified in two studies.^71,73^ Choudhary et al. and Upadhyay et al. both reported that Islamic religion and young maternal age (<20 years of age) were associated with lower odds of full immunization and higher odds of delayed vaccination for DPT–1. Female sex of the infant, birth weight <2,000 g, delivery by unskilled personnel, higher number of children and a lack of awareness about vaccination risks/benefits among mothers were also associated with lower odds of full immunization. In contrast, a high level of maternal education was strongly associated with improved vaccination status of the infant.^71,73^ Across studies, the main reason for a delay in vaccination was the general lack of awareness among HCPs and parents about vaccination benefits and concerns about possible adverse events due to vaccination in preterm and LBW infants.^71–73,76^ To this, we suggest the use of vaccines with published efficacy and safety data in the preterm and LBW infant population (Table 1).

Despite the availability of evidence and clear guidelines related to vaccination in India,^21,22^ there are wide knowledge gaps among HCPs and parents regarding the safety and efficacy of vaccines.^53^ Further details can be seen in Figure 1B. Several factors were found to influence the attitude of HCPs toward vaccination for preterm and LBW infants. These include the perception of limited vaccine effectiveness, the risk of vaccination-induced serious adverse events and contraindication following postnatal steroid administration.^56^ HCPs further perceive that birth weight, current weight, or the level of prematurity should determine the initiation of immunization.^50^ The lack of clear vaccination recommendations from HCPs ultimately guides the decision of parents or caregivers of the infant to reject vaccinations.^77^ Even if there are clear recommendations, a low education level and awareness of the parent or caregiver could delay vaccination or lead to refusal.^72,76,77^ In India, a lack of education for girls and young women, who are socially viewed as the primary caregiver, could undermine immunization efforts.^76^ Other factors such as home births in India^71^ and the cost of vaccination^73^ also tend to qualify as impediments to the vaccination of preterm and LBW infants.

## Strategies to mitigate the burden of VPDs in preterm and LBW infants

Successful treatment of infections in preterm and LBW infants relies on early recognition and diagnosis, which is known to be challenging.^70^ While the majority of infants will have some risk factors, there are several presenting symptoms that are nonspecific.^70^ Therefore, it has become imperative to prevent the burden of infectious diseases in preterm and LBW infants. This can be achieved using a two-fold strategy targeting both mothers and their newborn infants.

It is vital to reduce the risks of infection in premature infants through prevention of infections in expectant mothers. It is essential to implement a comprehensive strategy comprising multiple elements such as improving maternal nutritional status, diagnosing and treating pregnancy-related conditions, and providing adequate maternal and perinatal care. The prevention of infections through immunization activities, which are known to be effective in circumventing the risks associated with VPDs, should also be encouraged.^24^ Several immunization strategies have been suggested for the protection of newborn infants, the features of which are further discussed.

## Indirect immunization strategies

The use of indirect immunization strategies such as maternal immunization and cocooning have been suggested as relevant strategies to alleviate the burden of VPDs in infants (e.g. tetanus, pertussis, influenza etc.).^24,63–65,78^ Vaccination during pregnancy (maternal immunization) can provide protection against VPD for the mother, the developing fetus and the newborn through maternal antibodies transfer via the placenta and subsequently the breast milk.^79^ An example is neonatal tetanus, which tends to occur during the first 3–14 days of life and which carries a case fatality rate of 100% in newborns. Through immunization efforts, maternal and neonatal tetanus have been eliminated from India.^80^ Pertussis and influenza are other preventable diseases with potentially severe consequences (such as apnea, pneumonia and seizures in newborns) that can be averted through maternal immunization.^78,81^ Maternal immunization provides clear benefits. It is worth noting that the uptake of maternal immunization can however be slow.^82,83^ Common reasons include issues of confidence (i.e., fear of adverse pregnancy outcomes, lack of awareness, failure of the HCP to recommend vaccination and convenience/access [including cost]) and vaccine efficacy, driven possibly by the timing of vaccination.^82–84^

Recent studies have suggested that antigen‐specific cord-blood antibody titers are greater following maternal immunization with the tetanus, diphtheria, and acellular pertussis vaccine in the second, rather than the third trimester.^85,86^ For influenza vaccination, researchers have shown that seasonal influenza vaccination should be given at any stage of pregnancy, with the caveat that it takes 2 weeks after vaccination for the mother to be protected against influenza.^87–90^ Public health authorities have also revised their recommendations, with a few of them even recommending vaccinations as early as possible during pregnancy.^89,90^ Further research efforts to establish the appropriate timing of vaccinations during pregnancy could strengthen the use of maternal immunization in preventive neonatology.^84^

Other indirect immunization strategies such as cocooning could be considered when maternal immunization is missed or delayed. The IAP recommendation states that immunizing individuals who have regular contacts with a newborn might help reduce the risk of infection in newborns.^78^ However, there is little evidence to support the use of this strategy in protecting the extremely preterm and LBW infants. Additionally, cost and logistical barriers could further limit the widespread implementation of this strategy.^91,92^

## Direct immunization of preterm and LBW infants

In preterm and LBW infants, implementing the same vaccination schedule as set forth for full-term and normal birth weight infants appears crucial, as can be seen in the vaccination recommendations (Table 1). Specific guidance regarding the implementation of vaccination when the infant is in the neonatal intensive care unit (NICU) is not explicitly mentioned in the guidelines; there is limited evidence to suggest that vaccination could be considered in the NICU if the infant is stable or after discharge from the NICU in the ward.^93^ Table 1 also provides an overview of the main references that provide immunogenicity/efficacy, effectiveness and safety data for the recommended vaccinations specific to the preterm and LBW infant population. This evidence base supports the vaccination of infants regardless of prematurity level or birth weight at the recommended chronological age according to the vaccine-specific prescribing information.

Across the different vaccinations, the degree of immune response may vary in terms of geometric mean titers in preterm infants, but protective and durable responses are achieved in most cases.^94,95^ Studies have shown that, following administration of vaccines, preterm and LBW infants mount an immune response directly proportional to their gestational age and birth weight.^96^ Importantly, vaccines display a good safety profile even when given in combination, without compromising the immune response; this could potentially alleviate concerns of parents or HCPs with respect to safety.^97^ In addition, vaccinations recommended for use in healthy infants and children have shown good levels of efficacy, safety, and effectiveness regardless of prematurity or birth weight (Figure 1B).

Among the combination vaccines available, the diphtheria, tetanus, pertussis, hepatitis B, inactivated polio vaccine and *Hemophilus influenza*e type b (DTPa-HBV-IPV/Hib), given alone or with other pediatric vaccines, has a clinically acceptable safety and immunogenicity profile in preterm (>24 weeks) and LBW (as low as 700 g) infants as in full-term infants, although HBV and Hib vaccine responses appeared lower in preterm and LBW infants.^37^ The occurrence of post-immunization cardiorespiratory events is influenced by the severity of underlying neonatal conditions, but most tend to resolve spontaneously or require minimal intervention.^37^ These data make a strong case for the vaccination of preterm and LBW infants according to the schedule proposed for full-term and normal birth weight infants (i.e., chronological age). However, monitoring of the preterm/LBW infant up to 72 hours after vaccination is recommended.^98^ Notably, additional doses of HBV should be administered in infants receiving the first dose during the first days of life if they weigh less than 2,000 g because of a reduced immune response; for preterm infants born to hepatitis B Ag-positive mothers, both Ig and HBV should be given within 12 hours.^24,31,99^ The timeliness of vaccination and completion of the primary vaccination series at chronological age rather than gestational age appears crucial to provide the earliest possible protection in preterm and LBW infants.^95^ Importantly, we suggest the use of vaccines that have been tested in the preterm and LBW infant population and have robust efficacy and a clinically acceptable safety profile.

## Discussion

The considerations presented in this review have both clinical and public health implications for India. In recent decades, India has seen a signiﬁcant improvement in neonatal and infant health after the introduction of several initiatives by the Government of India (GOI).^51^ India’s National Health Policy 2017 set a target of 16 deaths per 1,000 live births for neonatal mortality by 2025,^100^ and the GOI has also set a target of less than 10 neonatal deaths per 1,000 live births by 2030 under the India Newborn Action Plan.^101^ Within this context, prematurity and LBW in neonates deserve special attention, as a significant number of children born in India are born preterm or have LBW.^14,19^ Although a systematic literature search was not included in this review, which is a limitation, it reaches its objective of raising awareness on the importance of reducing the incidence of VPDs in preterm and LBW infants in India through immunization.

Published evidence from studies conducted outside India indeed shows that prematurity and LBW can predispose the infant, given their immunocompromised status, to a high risk of VPDs.^54,56,69,96,102^ Reducing the incidence of VPDs in this vulnerable population after birth is the need of the hour. This can be achieved through timely immunization of the mother and newborn. Maternal immunization should be encouraged and there is a large evidence base supporting the safety and effectiveness of immunization during pregnancy.^84,103^ Similarly, vaccines in preterm and LBW infants are equally safe, immunogenic and effective as compared to full-term and normal birth weight infants.^94–96^ Generating more evidence on the timing of maternal immunization, as well as identifying and addressing barriers to vaccination uptake, are key challenges to overcome.^84,88^

In India, healthcare institutions advocate that preterm and LBW infants are vaccinated following the same schedule as that of their counterparts who are born full-term with normal birth weights, apart from the hepatitis B vaccine wherein an additional dose is required.^21–23^ Notwithstanding these recommendations, studies from India show that preterm and LBW infants are vaccinated with a significant delay,^71,73,76^ driven by the clinical judgment of the treating HCP whose recommendation is instrumental in ensuring vaccination. Delays due to true contraindications (e.g., severe combined immunodeficiency disease) are justified, but avoiding risks related to ‘small for gestational age’ or birthweight are often cited as the reason behind vaccination delays. LBW appears to be a strong indicator of vaccination delay. Given that being born preterm is a leading cause of LBW, gestational age could also be recognized as a predictor of vaccination delay.^50^ Data specific to vaccination delays in premature infants from India are lacking and are needed to shape the national vaccination policy. In addition, information assessing the relationship between vaccination delay and disease occurrence should be generated through large-scale observational studies. Further studies estimating vaccination coverage in preterm and LBW infants might provide insights on the scale of the problem and the underlying reasons for vaccination delay.

Delayed vaccination increases the susceptibility window to VPDs and their complications.^50^ There are several barriers in achieving timely vaccination of preterm and LBW infants in India. Among these, HCP and parent knowledge, perceptions and attitudes to vaccination stand out. The role of HCPs in facilitating immunization uptake is well-documented hence training HCPs to discuss the risks versus benefits of vaccinations with parents, on scientifically validated grounds, seems highly relevant.^50,77^ To achieve this, HCPs must regularly acquire up-to-date information on vaccinations in preterm and LBW infants. Besides efficacy and safety, parents tend to worry about the number of vaccinations.^53^ Targeted education and awareness initiatives for HCPs and health literacy interventions for parents, with focus on the importance, effectiveness and safety of vaccinations could help bridge immunization gaps in the vulnerable preterm and LBW infant population. In addition, the use of combination vaccines should be encouraged, as it addresses parents’ fears of multiple injections and increases the acceptance and compliance with the vaccination schedule.^97^

## Conclusion

Routine childhood vaccinations can help reduce or eliminate the burden of VPDs and should be given to preterm and LBW babies, regardless of prematurity or birth weight. It is crucial that HCPs are made aware that preterm and LBW infants could be faced with detrimental health effects if vaccinations are not administered in a timely manner. Inappropriate delays in vaccinating this fragile population should be minimized by ensuring that vaccination discussions are encouraged with families and caregivers at the point of care. These steps should be closely integrated within neonatal and other overall infant health management strategies to increase vaccination compliance and improve health in the fragile population of preterm and LBW infants.
